# Molecular characterization and immune efficacy of fructose-1,6-bisphosphate aldolase from *Haemaphysalis longicornis* (Acari: Ixodidae)

**DOI:** 10.1186/s13071-023-05794-1

**Published:** 2023-05-25

**Authors:** Yuan-Yuan Cao, Shu-Wen Xiao, Feng Yang, Xiao-Ya Liu, Hui Lu, Jin-Cheng Zhang, Yong-Hong Hu

**Affiliations:** 1 Ministry of Education Key Laboratory of Molecular and Cellular Biology, Hebei Key Laboratory of Animal Physiology, Biochemistry and Molecular Biology, Hebei Collaborative Innovation Center for Eco-Environment, College of Life Sciences, Hebei Normal University, Shijiazhuang, 050024 China; 2 Shijiazhuang Post and Telecommunication Technical College, Shijiazhuang, 050021 China

**Keywords:** Tick, Fructose-1,6-bisphosphate aldolase, Immune efficacy

## Abstract

**Background:**

Ticks are obligate hematophagous ectoparasites that transmit a variety of pathogens to humans, wildlife and domestic animals. Vaccination is an effective and environmentally friendly method for tick control. Fructose-1,6-bisphosphate aldolase (FBA) is an important glycometabolism enzyme that is a candidate vaccine against parasites. However, the immune protection of FBA in ticks is unclear.

**Methods and results:**

The 1092-bp open reading frame (ORF) of FBA from *Haemaphysalis longicornis* (HlFBA), encoding a 363-amino acid protein, was cloned using PCR methodology. The prokaryotic expression vector pET32a(+)-HlFBA was constructed and transformed into cells of *Escherichia coli* BL21(DE3) strain for protein expression. The recombinant HlFBA protein (rHlFBA) was purified by affinity chromatography, and the western blot results suggested that the rHlFBA protein was immunogenic.

**Results:**

Results of the enzyme-linked immunosorbent assay showed that rabbits immunized with rHlFBA produced a humoral immune response specific to rHlFBA. A tick infestation trial indicated that, compared to the ticks in the histidine-tagged thioredoxin (Trx) group, the engorged tick weight and oviposition of female ticks and egg hatching rate of those in the rHlFBA group was reduced by 22.6%, 45.6% and 24.1%, respectively. Based on the cumulative effect of the these three parameters, the overall immune efficacy of rHlFBA was estimated to be 68.4%.

**Conclusions:**

FBA is a candidate anti-tick vaccine that can significantly reduce the engorged tick weight, oviposition, and egg hatching rate. The use of enzymes involved in glucose metabolism is a new strategy in the development of anti-tick vaccines.

**Graphical Abstract:**

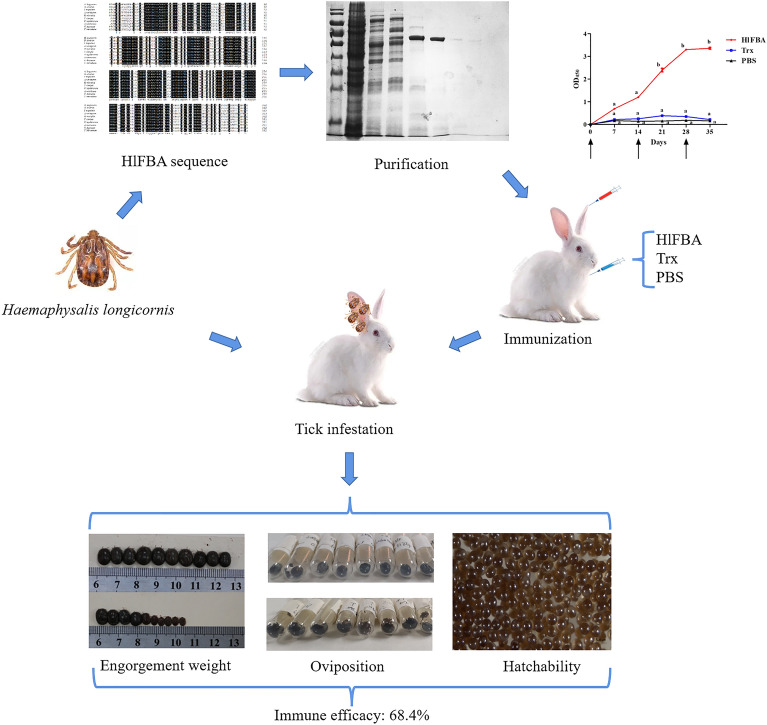

## Background

Ticks, which are obligatory blood-feeding arthropods, are major pathogen vectors in humans and animals worldwide [1]. Both ticks and the microbes they transmit are significant threats to human and veterinary health [2]. *Haemaphysalis longicornis* (Acari: Ixodidae) is a tick species native to Eastern Asia and has become established in Australia, New Zealand and several Pacific islands [3]. It transmits pathogens such as *Theileria uilenbergi*, *Babesia motasi*, *Rickettsia hebeiii* and *Anaplasma phagocytophilum* [4–7]. It is also the vector of severe fever with thrombocytopenia syndrome virus (SFTSV), which endangers human and animal health [8]. Therefore, it is imperative to develop an anti-*H. longicornis* vaccine [9, 10].

Galay et al. evaluated two kinds of the iron-binding protein ferritins from *H. longicornis* (HlFER), an intracellular HlFER1 and a secretory HlFER2, as anti-tick vaccines [11]. Western blot results demonstrated that antibodies cross-reacted with the recombinant HlFER (rHlFER) and also reacted with native HlFERs. A tick challenge experiment demonstrated that ticks fed on the rHlFER2-inoculated rabbit had lower engorgement weight than ticks in the control group. Oviposition and hatchability were reduced in both the rHlFER-inoculated groups. The vaccine efficacy of rHlFER1 and rHlFER2 was 34% and 49%, respectively [11]. In another study, the open reading frame (ORF) of *H. longicornis* subolesin (HlSu) was identified, and the recombinant HlSu (rHlSu) expressed in *Escherichia coli* [12]. Rabbits immunized with rHlSu produced an immune response. In the rHlSu-immunized group, engorgement weight and oviposition of female ticks were significantly lower than in the control group. The calculated vaccine efficacy was estimated to be 37.4% [12]. In a subsequent study, Wang et al. cloned the *H. longicornis* lipocalin homolog (HlLIP) gene into pET-32(a^+^) to obtain the recombinant protein (rHlLIP); the immunogenicity of RHlLIP was confirmed by western blot [13]. An immunization trial on rabbits infested with *H. longicornis* showed that antibodies against the rHlLIP protein reduced engorgement weight, oviposition and hatchability (vaccine efficacy of the rHlLIP protein was 60.17%). However, to date, a commercial anti-*H. longicornis* vaccine is not currently available, so it is important to screen for an efficient protective antigen against *H. longicornis* infestation.

Fructose-1,6-bisphosphate aldolase (FBA) is an enzyme that catalyzes fructose-1,6-bisphosphate to glyceraldehyde-3-phosphate and dihydroxyacetone phosphate or the reverse aldol alcohol condensation reaction [14]. FBA, which can induce immune responses in many infectious diseases [15], can also be used as a broad-spectrum vaccine against many pathogens [16–20]. In one study, mice inoculated with recombinant FBA (rFBA) + Freund's adjuvant acquired immune protection against *Streptococcus pneumoniae* challenge [21]. Compared with Freund’s adjuvant alone, the rFBA + Freund’s adjuvant increased the proliferation of memory CD4^+^ T cells in mice and significantly increased survival rates. Mice anti-rFBA sera also significantly protected the mice against a lethal *S. pneumoniae* challenge compared to preimmune sera [21]. These data suggest that rFBA is a candidate vaccine for protection against *S. pneumoniae.* McCarthy et al. verified that *Onchocerca volvulus* FBA (OvFBA) is immunogenic. When the recombinant OvFBA was tested for protective efficacy in mice, larval survival was reduced by 50% [16]. These data support further study of this enzyme as a candidate vaccine in animal models.

In the present study, we cloned the ORF of FBA from *H. longicornis* (HlFBA) and used *E. coli* to express the recombinant HlFBA protein (rHlFBA). We then analyzed its immune protection efficiency by immunizing rabbits. The results indicate that H1FBA could be a useful vaccine against *H. longicornis* infection.

## Methods

### Ticks and rabbits

Adult ticks of *H. longicornis* were collected from sheep of the Hebei Province Xiaowutai National Natural Reserve Area in May and sent to the laboratory as previously described [22]. During the non-parasitic phase, ticks were maintained in an incubator; during the parasitic phase, ticks were fed on the ears of New Zealand white rabbits. The next generation of adult ticks was collected at different developmental stages for the experiments. All animal experiments were performed according to approved protocols of the Animal Ethics Committee of Hebei Normal University (#2021LLSC035).

### Full-length sequence amplification of *HlFBA*

Total RNA from *H. longicornis* adult females was extracted using TRIzol (TransGen, Beijing, China) reagent for further complementary DNA (cDNA) synthesis as previously described [23]. The ORF of the H1FBA gene (*HlFBA*) was amplified and cloned using primers 5′-ATGGCTGGCCACTTCAC-3′ and 5′-TCAGTACTCGTGGTTTTTGATA-3′. *HlFBA* was obtained by PCR cycling consisting of the following steps: pre-degeneration at 94 °C for 3 min; 30 cycles of denaturation at 94 °C for 30 s, annealing at 60 °C for 30 s and extension at 72 °C for 60 s; a final extension at 72 °C for 10 min. The PCR products obtained were separated by agarose gel electrophoresis, followed by the recovery and ligation of the target bands into a pEASY-T1 vector, which was subsequently transformed into TRANS-T1 cells (TransGen) for sequencing. The correct gene sequence was translated into the amino acid sequence followed by multiple sequence alignment using DNAMAN 8.0 (Lynnon Biosoft, Quebec, ONT, Canada).

### Analysis of *HlFBA* expression level

The transcription profiles of *HlFBA* from different developmental stages (egg, larva, nymph and adult) and different organs (salivary glands, midgut, ovary and malpighian tubules) of female ticks were estimated by quantitative real-time PCR (qRT-PCR). Specific primers of *HlFBA* were designed (forward primer: 5′-TCTGACCAAGAGGTGCGT-3′; reverse primer: 5′-GTAGCGAGCGAGGAC-ATT-3′). Relative expression of the *H. longicornis* β-actin gene (AN AY254898) was used to normalize the *HlFBA* expression data [24]. The gene expression data were calculated by the 2^−ΔΔCt^ method [25]. All analyses were performed using three technical and three biological replicates.

### Expression and purification of the rHlFBA protein

The primers containing *Eco*RI and *Hin*dIII restriction sites (forward primer: 5′-GAATTCATGGCTGGCCACTTCAC-3′; reverse primer: 5′-AAGCTTTCAG-TACTCGTGGTTTTTGATA-3′) were used to amplify *HlFBA* by PCR. The PCR products were separated by agarose electrophoresis, and the target gene was recovered using a DNA gel recovery kit (BioTeke Corp., Beijing, China) and inserted into the prokaryotic expression plasmid pET-32a(+) (TransGen), named pET-32a(+)-HlFBA. For expression of rHlFBA, plasmids were transformed into cells of *E. coli* strain BL21(DE3) (Takara Bio Inc., Shiga, Japan) and the cells propagated in 10 ml Luria–Bertani (LB) broth containing 10 μg/ml ampicillin (TransGen) overnight at 37 °C and 200 rpm, following which they were then used to inoculate 100 ml cultures. The rHlFBA protein was induced by 0.5 mM isopropyl-β-d-1-thiogalactopyranoside (IPTG) at 25 °C, and the supernatants of the cell cultures were purified by elution along an imidazole gradient (20 mM, 50 mM, 100 mM, 200 mM and 500 mM) in Ni. Sepharose 6 Fast Flow chromatography resin (GE Healthcare, Chicago, IL, USA). The rHlFBA protein was quantified using the Bradford method [26]. The histidine-tagged thioredoxin (Trx) protein expressed by plasmid pET-32(a^+^) was purified using the above procedure.

### Western blot analysis

Ten unfed female ticks were ground in liquid nitrogen and transferred to a tube containing 1 ml 0.1 M phosphate-buffered saline (PBS). After centrifugation at 13,000 rpm for 10 min at 4 ˚C, 500 μg of tick protein, mixed with Freund’s complete adjuvant, was injected into a rabbit. This was followed by two injections of 500 μg of tick protein mixed with Freund’s incomplete adjuvant at intervals of 2 weeks. Sera were collected at day 14 after the last immunization and purified by the caprylic acid-ammonium sulphate precipitation method to obtain rabbit anti*-H. longicornis* serum.

A 20-μg sample of total protein, including prestained protein marker (TransGen), purified rHlFBA and IPTG-induced *E. coli* containing pET-32a(+)-HlFBA, was loaded onto 12% sodium dodecyl sulfate-polyacrylamide gel electrophoresis (SDS-PAGE) gels, stained with Coomassie Brilliant Blue and transferred to polyvinylidene difluoride (PVDF) membranes. After blocking in TBS-Tween-20 (TBST) containing 5% fat-free milk at 25 °C for 3 h, the membrane was incubated with 1:2000 diluted rabbit anti-*H. longicornis* serum or rabbit negative serum overnight, respectively. The membranes were then washed with TBST and incubated with 1:2000 diluted horseradish peroxidase (HRP)-conjugated goat anti-rabbit immunoglobulin G (IgG; Proteintech, Chicago, IL, USA) for 2 h. Positive signals were detected using SuperSignal® West Dura Extended Duration Substrate (Thermo Fisher Scientific, Waltham, MA, USA) and photographed through an chemiluminescence imaging system (Bio-Rad Laboratories, Hercules, CA, USA).

### Rabbit immunization

Rabbits were randomly divided into the PBS group, Trx group and rHlFBA group (6 rabbits per group). In the experimental group, 0.5 ml rHlFBA (1 μg/μl) mixed with 0.5 ml Freund’s complete adjuvant was injected into the back of rabbits at day 0, and the same dose of rHlFBA mixed with Freund’s incomplete adjuvant was injected into the back of rabbits at day 14 and day 28, respectively. In the control group, 0.5 ml PBS or Trx protein (1 μg/μl) mixed with adjuvant was injected using the same protocol into the back of rabbits.

### Antibody level determination by enzyme-linked immunosorbent assay

Before the first immunization and on days 7, 14, 21, 28, and 35 after the first immunization, blood was collected from the ears of rabbits for antibody level analysis and determination of the optical density (OD) values; measurements were made at the same dilution, reflecting the antibody level [27]. The rHlFBA proteins (1 μg/well) were used to coat enzyme-linked immunosorbent assay (ELISA) plates overnight at 4 °C, following which the plates were blocked with bovine serum albumin at 37 °C for 1 h. The plates were then incubated with the immunized-rabbit sera at 37 °C for 1 h, which had been diluted serially from 1:200 to 1:204,800, and then with HRP-conjugated goat anti-rabbit IgG (1:10,000) for 1 h at 37 °C, respectively. Finally, 3,3′, 5,5′-tetramethylbenzidine as color-substrate solution was added to the plates and the reaction was terminated with the addition of sulfuric acid solution. The absorbance at OD_450_ was measured by using a microplate reader (Molecular Devices, Silicon Valley, CA, USA).

### Tick infestation trial

On the 10th day after the third immunization of rabbits (3 groups, 6 rabbits per group), 46 adult ticks (23 females and 23 males) were released into cloth bags glued onto one ear of the rabbit. The same treatment was performed on the other ear of the rabbit. Then, after the detachment of female ticks from hosts, the number of biting ticks, engorged tick weight, oviposition and egg-hatching rates were recorded. Vaccine efficacy (E) was calculated as 100 × [1–(E_W_ × E_O_ × E_H_)], where E_W_, E_O_ and E_H_ represent engorged tick weight, oviposition and egg-hatching rates from the experimental groups/control group, respectively [11].

### Statistical analysis

Statistical analyses were conducted using SPSS version 17.0 software (IBM Corp., Armonk, NY, USA). All data presented in figures and tables were checked for normality. Antibody levels in blood collected from the different groups at the same time point were analyzed by ELISA to determine differences between the experimental group and the control group by one-way analysis of variance (ANOVA). The expression of *HlFBA* in the qRT-PCR analysis and the different parameters (engorgement weight, oviposition and hatchability) in the tick infestation trial were analyzed by one-way ANOVA followed by the Tukey test, to determine the differences between the different treatments. The significance level was set at *P* < 0.05.

## Results

### Gene cloning and alignment of HlFBA

**Fig. 1:**
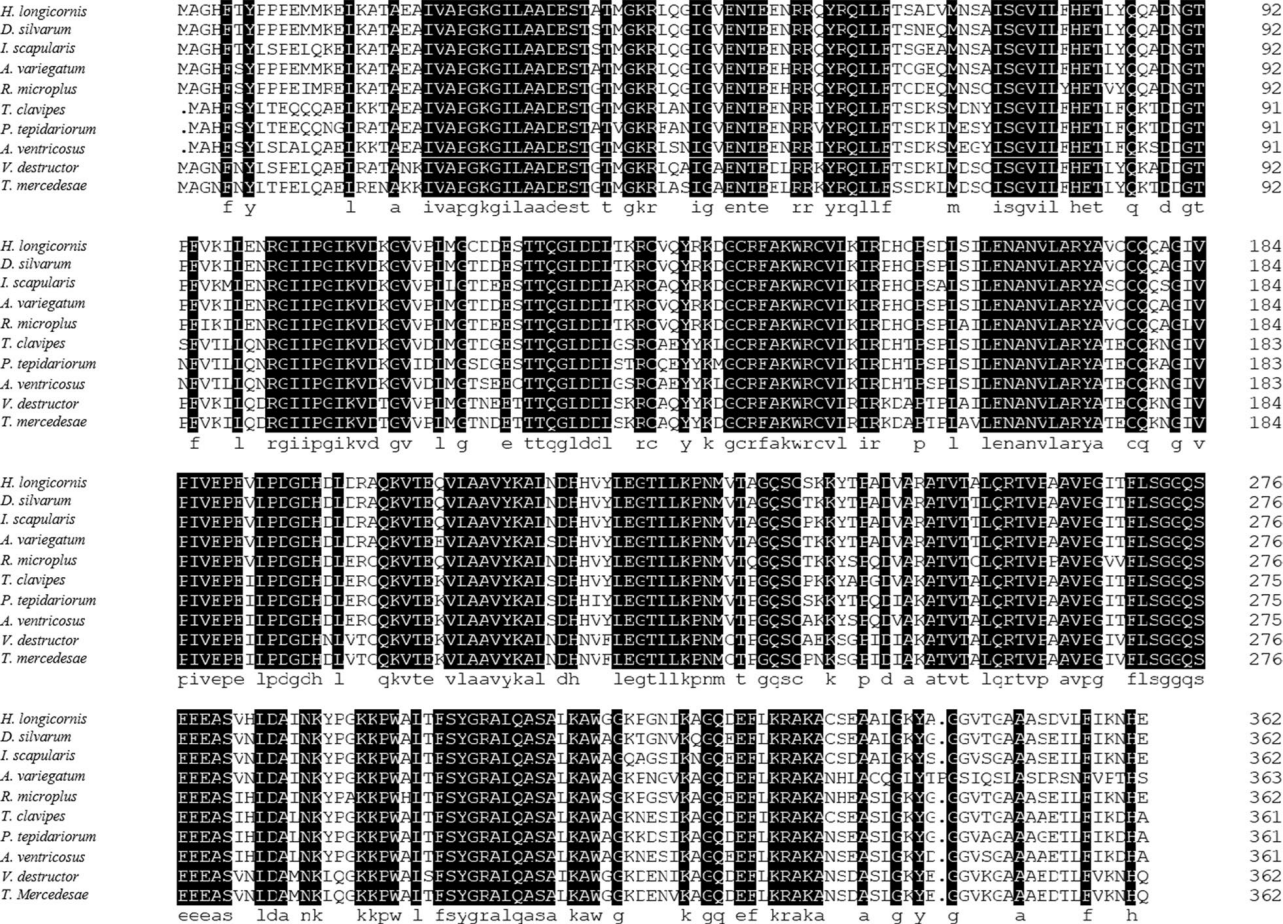
Alignment of predicted amino acid sequences of fructose-1,6-bisphosphate aldolase protein from *Haemaphysalis longicornis* and other species of Arachnida. GenBank accession numbers: *H. longicornis* (ASV64058.1), *Dermacentor silvarum* (XP_037566647.1), *Ixodes scapularis* (XP_029847818.1), *Amblyomma variegatum* (DAA34561.1), *Rhipicephalusmicroplus* (XP_037283106.1), *Trichonephila clavipes* (GFY24485.1), *Parasteatoda tepidariorum* (XP_042900530.1), *Araneus ventricosus* (GBN85837.1), *Varroa destructor* (XP_022668058.1) and *Tropilaelaps mercedesae* (OQR70008.1)

The length of the ORF from *HlFBA* in *H. longicornis* was 1092 bp (KX839690.1) and encoded 363 amino acids (aa). Multiple alignment results indicated that the HlFBA protein (ASV64058.1) shared 95%, 91%, 90% and 89% similarity with FBA from *Dermacentor silvarum* (XP_037566647.1), *Ixodes scapularis* (XP_029847818.1), *Amblyomma variegatum* (DAA34561.1) and *Rhipicephalus microplus* (XP_037283106.1), respectively. The HlFBA protein had 77%–81% conserved amino acids compared to the FBAs of other arachnids, such as *Araneus ventricosus* (GBN85837.1), *Parasteatoda tepidariorum* (XP_042900530.1), *Varroa destructor* (XP_022668058.1), *Trichonephila clavipes* (GFY24485.1) and *Tropilaelaps mercedesae* (OQR70008.1) (Fig. 1).

### qRT-PCR detection of *HlFBA*

**Fig. 2:**
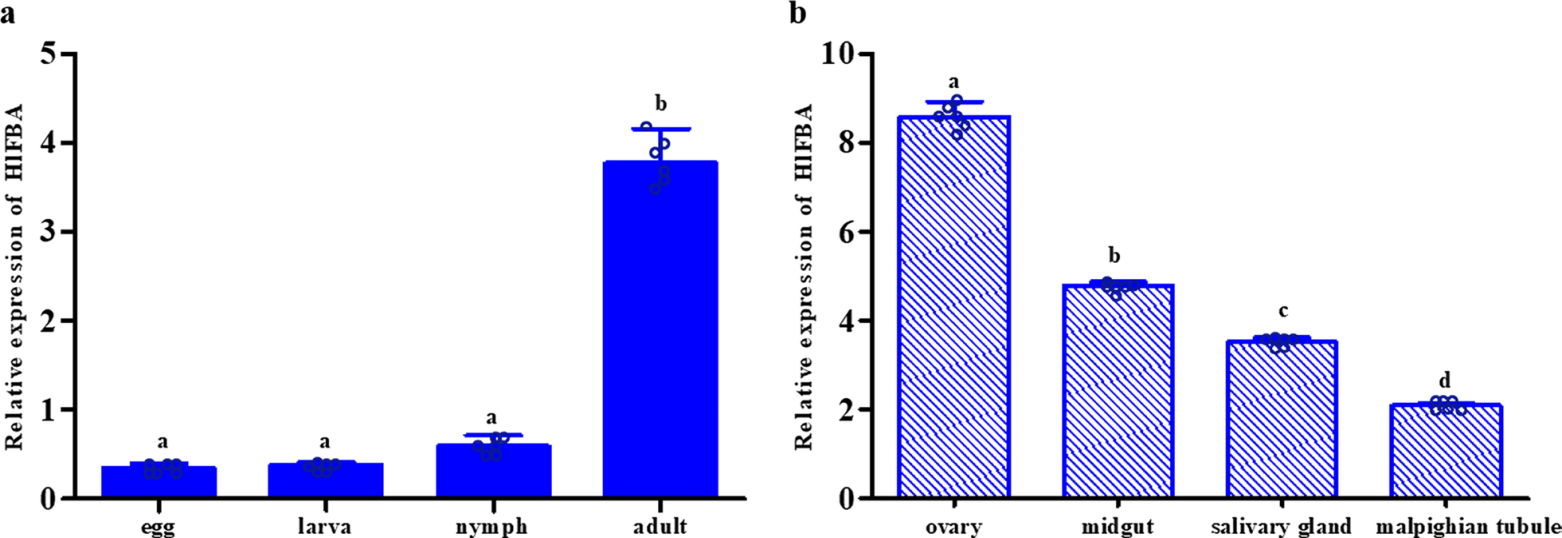
Expression patterns of *HlFBA* in different developmental stages (**a**) and different organs (**b**). Different lowercase letters above bars represent significant differences across the group (*P* < 0.05). Circles represent individual points of each group. All analyses were performed using three technical and three biological replicates.* HlFBA*, Fructose-1,6-bisphosphate aldolase gene from *Haemaphysalis longicornis*

The qRT-PCR results showed that *HlFBA* was expressed in different developmental stages and that its expression in adult ticks was significantly higher than that in other developmental stages (*F* = 7.196;* df* = 3, 8; *P* < 0.01; Fig. 2a). Moreover, *HlFBA* was found in all detected tissues of female ticks, with the ovary showing the highest expression level (*F* = 218.197;* df* = 3, 8; *P* < 0.01; Fig. 2b).

### Production and western blot of rHlFBA protein

**Fig. 3:**
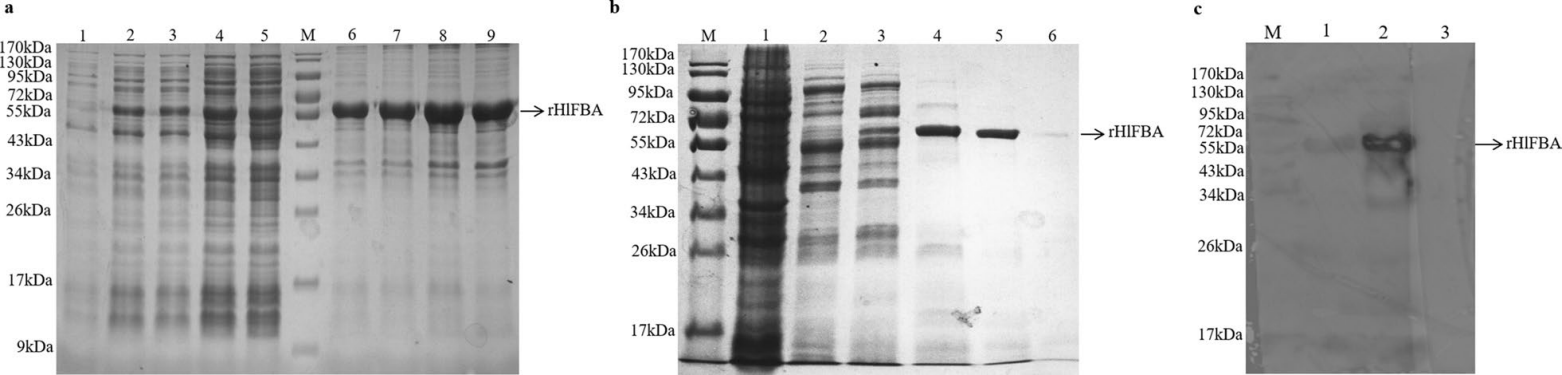
Sodium dodecyl sulfate–polyacrylamide gel electrophoresis (SDS-PAGE) and western blot analysis of rHlFBA. **a **SDS-PAGE of rHlFBA protein expression in cells of *Escherichia coli* BL21 strain induced by 0.5 mM isopropyl-β-d-1-thiogalactopyranoside (IPTG) at 25 °C. Lanes:* 1* Production of the rHlFBA protein without IPTG;* 2*,* 3*,* 4*,* 5* production of rHlFBA protein in the supernatant with IPTG at 25 °C for 2, 4, 6 and 8 h, respectively;* M* marker;* 6*,* 7*,* 8*,* 9* production of the rHlFBA protein in the precipitation with IPTG at 25 °C for 2, 4, 6 and 8 h, respectively. **b** SDS-PAGE analysis of rHlFBA protein elution. Lanes:* M* Marker;* 1* production of the rHlFBA protein with IPTG induction;* 2–6* rHlFBA protein eluted with 20 mM, 50 mM, 100 mM, 200 mM and 500 mM imidazole, respectively. **c** Western blot analysis of the rHlFBA protein. Lanes:* M* Marker;* 1* purified rHlFBA by Ni column incubated with rabbit anti-*H. longicornis* serum;* 2* IPTG-induced *E. coli* with pET-32a(+)-HlFBA incubated with rabbit anti-*H. longicornis* serum;* 3* purified rHlFBA by Ni column incubated with rabbit negative serum. Arrows indicate the rHlFBA protein. rH1FBA, Recombinant fructose-1,6-bisphosphate aldolase from *Haemaphysalis longicornis*

The molecular weight of rHlFBA protein was about 62 kDa, as determined by SDS-PAGE, with Trx from the empty plasmid accounting for 22 kDa (Fig. 3a). The rHlFBA protein was highly expressed in the supernatant induced for 6 h at 25 °C and purified well under 200 mM imidazole elution conditions (Fig. 3b). The western blot results showed that only the rHlFBA protein reacted positively with rabbit anti-*H. longicornis* serum, thus indicating its immunogenic specificity (Fig. 3c).

### Analysis of antibody level by ELISA

**Fig. 4:**
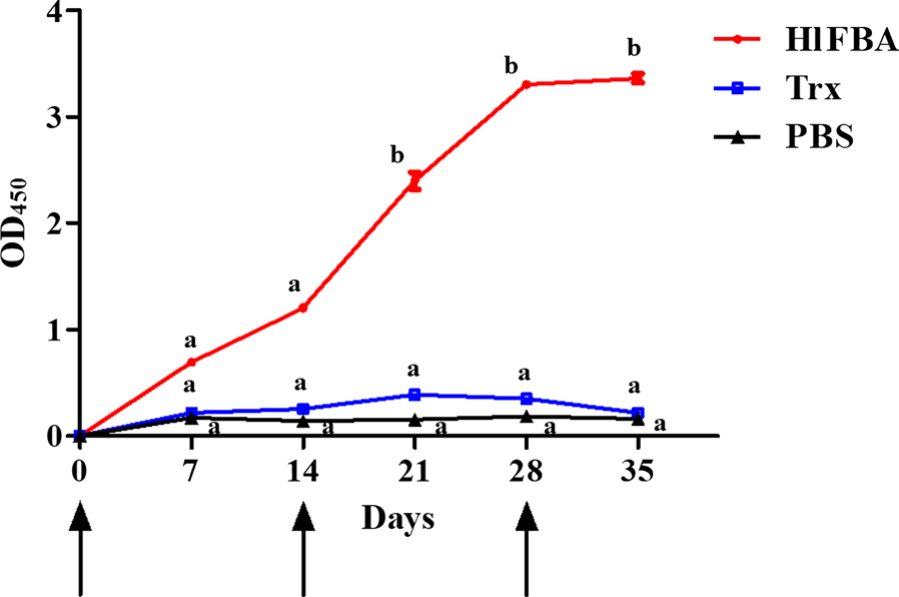
Detection of anti-rHlFBA (recombinant fructose-1,6-bisphosphate aldolase from *Haemaphysalis longicornis*) antibody level in rabbit serum by enzyme-linked immunosorbent assay. Arrows indicate the days of immunization days. Different lowercase letters represent significant differences across the group at the indicated time point (*P* < 0.05). Red circles, blue squares and black triangles represent the OD values detected by ELISA in the HlFBA group, Trx group, and PBS group at different time points, respectively. All analyses were performed using three technical and three biological replicates

The ELISA results showed that the antibody level of the rabbits immunized with rHlFBA gradually increased with increasing immunization time. The antibody level began to increase significantly on the 7th day after the second inoculation (*F* = 677.175;* df* = 2, 3; *P* < 0.01) and maintained a higher level until the third inoculation (Fig. 4). However, antibody levels in the Trx group and PBS group did not change significantly (*F* = 677.175;* df* = 2, 3; *P* = 0.081).

### Tick infestation trial

On the 10th day after the third immunization, New Zealand white rabbits were challenged with adult ticks. The different parameters of the PBS group, Trx group and rHlFBA group were analyzed by one-way ANOVA. The results of the tick infestation trial suggested that engorgement weight, oviposition and hatchability between the PBS group and Trx group did not change significantly over time (*F* = 5.258, *P* = 0.995;* F* = 9.331, *P* = 0.448;* F* = 80.895, *P* = 0.947, respectively;* df* = 2, 15 for all; Table 1). Compared with the Trx group, the mean (± standard error) engorged tick weight (*F* = 5.258;* df* = 2, 15; *P* < 0.05), oviposition (*F* = 9.331;* df* = 2, 15; *P* < 0.01) and egg-hatching rates (*F* = 80.895,* df* = 2, 15; *P* < 0.01) from the rHlFBA group were 142.10 ± 25.09, 36.12 ± 13.68 and 59.65 ± 1.86%, which were a reduction of 22.6%, 45.6% and 24.1%, respectively. The estimated immune efficiency, calculated according to the above parameters, of the vaccination with rHlFBA was 68.4%.

**Table 1:** Effect of vaccination with recombinant fructose-1,6-bisphosphate aldolase protein from *Haemaphysalis longicornis* on tick infestations

Trial	Total number of female ticks	Number of biting ticks	Engorgement weight (mg)	Oviposition (mg)	Hatchability (%)	E_W_^a^	E_O_^a^	E_H_^a^	E (%)^b^
Phosphate-buffered saline (PBS) group (*n* = 6)	276	188	193.56 ± 22.80a	73.52 ± 18.85a	78.44 ± 4.52a	–	–	–	
Thioredoxin (Trx) group (*n* = 6)	276	218	183.48 ± 19.41a	66.36 ± 14.76a	78.56 ± 2.77a				
rHlFBA group (*n* = 6)	276	213	142.10 ± 25.09b	36.12 ± 13.68b	59.65 ± 1.86b	0.77	0.54	0.76	68.4

Data on engorgement weight, oviposition and hatchability are presented as the mean ± standard error. Values followed by different lowercase letters in columns represent significant differences between the group (*P* < 0.05)

rHlFBA, Recombinant fructose-1,6-bisphosphate aldolase from *Haemaphysalis longicornis*

^a^Formulas for the calculation of reduction in engorgement weight (E_W_), oviposition (E_O_) and hatchability (E_H_) are described in the Methods section

^b^Efficacy (E) = 100 [1- (E_W_ × E_O_ × E_H_)]. The number of rabbits immunized in each group is six (*n* = 6)

## Discussion

Fructose-1,6-bisphosphate aldolase is a key enzyme in glycolysis and gluconeogenesis, and it is closely associated with life activities, energy acquisition and metabolic activities of organisms [14]. Several studies have shown that, in addition to its normal glycolytic role, it plays a role in host invasion [28]. In the present study, we performed molecular characterization of HlFBA from *H. longicornis* and estimated its efficacy in providing immune protection to rabbits.

Prompipak et al. amplified the FBA gene of *Opisthorchis viverrini* by PCR, and determined that its full-length sequence was 1089 bp, encoding 362 aa [15]. Li et al. characterized the FBA of *Clonorchis sinensis* FBA, reporting three ORFs (CsFBA-1, CsFBA-2 and CsFBA-3) with lengths of 1089, 1092 and 1092 bp, encoding 362 aa, 363 aa and 363 aa, respectively [29]. In the present study, the size of protein encoded by *HlFBA* from *H. longicornis* was consistent with those reported for parasitic trematode species. The sequence alignment showed that the identity of the amino acid sequence between HlFBA from *H. longicornis* and those from other ticks was > 89%, but < 81% with those from other species of Arachnida, which is consistent with their evolutionary relationship.

Yang et al. detected transcription of *Trichinella spiralis* FBA (TsFBA) messenger RNA (mRNA) at all developmental stages of *T. spiralis* [30]. In the present study, the qRT-PCR results showed that *HlFBA* expression occurred in different developmental stages of ticks, being highest in adults. This may be related to differences in individual size and metabolic activity. Adult ticks are larger, with greater metabolic activity and energy needs, so this may be the reason why expression is the highest in this stage. The difference in expression in different tissues is suitable for the physiological function of each tissue. The highest expression of *HlFBA* in the ovary may be related to vigorous metabolism and the highest metabolic activity in the early stage of egg formation. However, the specific mechanism needs further study.

As FBA plays a central role in parasite activities and survival, it has been considered to be a potential vaccine candidate or chemotherapeutic target for treatment [30]. In the study by Yang et al., after mice were immunized with the recombinant *T. spiralis* FBA (rTsFBA), the ELISA results showed a significant increase in the IgG levels in the rTsFBA group compared with the control group. This resulted in a T helper 1/T helper 2 (Th1/Th2) mixed humoral and cellular immune response, with Th2 cells being predominant, as well as highly elevated IgE levels [30]. In an other study, when mice were immunized with the recombinant FBA protein of *Schistosoma mansoni* they developed high levels of IgG or IgG1 [31]. A similar result occurred in the present study. Compared to the control group, the antibody level in the rHlFBA group increased significantly on the 7th day after the second inoculation until the third inoculation (*P* < 0.05). It can therefore be concluded that immunization with rHlFBA protein can induce rabbits to produce humoral immunity.

Other studies have also demonstrated the protective efficacy of FBA against various parasite challenges [31, 32]. Mice vaccinated with rTsFBA displayed a 48.7% reduction in adult worm burden and a 52.5% reduction in muscle larval burden [30]. These data showed that TsFBA is an effective antigen for developing a vaccine against *T. spiralis* infection. Marques et al. linked the gene of *S. mansoni* FBA into the pGEX-4 T-3 plasmid and its fusion protein was produced in *E. coli* [31]. Immunization of mice with this antigen induced significant protection (57%) against cercariae infection and hepatic granuloma formation decreased significantly [31]. The FBA from adult* S. mansoni* also reduces the formation of *S. mansoni* hepatic granulomas in immunized mice. FBA plays a central role in glycolysis and is important for the production of the energy required for different schistosome activities and survival and it therefore has become a target for intervention [33–35]. These findings support the use of FBA as a promising candidate vaccine against parasites. Our statistical analysis showed that the immune efficiency of rHlFBA was 68.4%. Compared with the control group, the engorged tick weight, oviposition and egg-hatching rates from the rHlFBA group significantly decreased by 22.6%, 45.6% and 24.1%, respectively (*P* < 0.05). An earlier study showed that antibodies in host blood can cross the midgut of *Rhipicephalus appendiculatus* and retain binding activity within the adult female [36]. It can be hypothesized that antibody may bind to the HlFBA protein in the tick and mediate the loss of FBA function. This would result in failure of the tick to obtain energy in a normal manner. This loss of energy could manifest by reduction of engorged tick weight, oviposition and egg hatching rates of *H. longicornis* ticks.

In an earlier study, we found that the immune protection of triosephosphate isomerase (TIM) in *H. longicornis* (HlTIM) was 50.8% [23]. The present study indicated that the vaccine efficiency of HlFBA (68.4%) is higher than that of HlTIM. Higher antibody titers are correlated with host protection against tick infestations [37]. This conclusion was confirmed by the higher antibody level of the HlFBA group from the results of the ELISA in our studies. Therefore, the reduction ratios of three parameters (engorgement weight, oviposition, hatchability) in the HlFBA group (22.6%, 45.6% and 24.1%) were higher than those of the HlTIM group (8.6%, 35.4% and 17.3%). Moreover, the physiological functions of FBA and TIM antigens were different, and the degree of function damage mediated by antibodies was different. Our results showed that the FBA gene is highly conserved in many tick species and that this would be beneficial for the development of a broad-spectrum anti-tick vaccine. The cross-reactivity of this protein with other tick-derived sera and related immunization experiments should be analyzed to evaluate its feasibility for vaccine development.

## Conclusions

The rHlFBA protein can particularly protect rabbits against *H. longicornis* infection. Vaccination with single-antigen FBA may inhibit the physiological responses in ticks, affect normal female tick development and protect the host. The study of enzymes involved in glucose metabolism could help drive the development of a broad-spectrum vaccine against ticks.

## Data Availability

The data supporting the conclusions of this article are included within the article.

## Abbreviations

ELISA: Enzyme-linked immunosorbent assays
FBA: Fructose-1,6-bisphosphate aldolase
HlFBA: FBA from *Haemaphysalis longicornis*
HRP: Horseradish peroxidase
IPTG: Isopropyl-β-d-1-thiogalactopyranoside
ORF: Open reading frame
PVDF: Polyvinylidene difluoride
qRT-PCR: Quantitative real-time PCR
rHlFBA: Recombinant HlFBA protein
TBST: TBS-Tween-20
Trx: Thioredoxin
